# Detection of *Mycoplasma pneumoniae* in hospitalized children with pneumonia in Laos

**DOI:** 10.12688/wellcomeopenres.19894.5

**Published:** 2025-08-07

**Authors:** Keoudomphone Vilivong, Mayfong Mayxay, David A.B. Dance, Xavier De Lamballerie, Paul N. Newton, Audrey Dubot-Pérès

**Affiliations:** 1 Lao-Oxford-Mahosot Hospital-Wellcome Trust Research Unit (LOMWRU), Microbiology Laboratory, Mahosot Hospital, Vientiane, Lao People's Democratic Republic; 2 Institute of Research and Education Development (IRED), University of Health Sciences, Ministry of Health, Vientiane, Lao People's Democratic Republic; 3 Centre for Tropical Medicine and Global Health, Nuffield Department of Medicine, University of Oxford, Oxford, England, UK; 4 Faculty of Infectious and Tropical Diseases, London School of Hygiene and Tropical Medicine, London, UK; 5 Unité des Virus Émergents (UVE: Aix-Marseille Univ-IRD 190-Inserm 1207), Marseille, France

**Keywords:** Mycoplasma pneumoniae, acute respiratory infection, paediatric, Laos, diagnosis

## Abstract

*Mycoplasma pneumoniae* has been described worldwide as an important cause of community-acquired pneumonia. From December 2013 to December 2014, 461 children admitted to Mahosot Hospital, Vientiane, Laos, with acute respiratory infection were investigated for upper respiratory microorganisms using probe-based real-time polymerase chain reaction (PCR) (FTD33). *M. pneumoniae* was detected by FTD33 in the upper respiratory tract of three patients, two girls and one boy, 5.7 and 3.9 years old and 13.6 years old, respectively. They presented with clinical features compatible with *M. pneumoniae* infection. They improved without *M. pneumoniae* directed therapy. The two girls were also positive for other potential pathogens. The boy had abnormal pulmonary auscultation, and one of the girls had significant anaemia. These results suggest that enhancement of diagnostic systems for *M. pneumoniae* detection is needed to improve understanding of the epidemiology of *M. pneumoniae* infection in Laos.

## Introduction

*Mycoplasma* spp., the smallest self-replicating microorganism, is associated with both upper and lower respiratory tract infections, most commonly tracheobronchitis, and diverse other clinical manifestations, especially of the skin and central nervous system. *M. pneumoniae* infection, which occurs worldwide with both endemic and epidemic patterns, may have been responsible for up to 20–40% of community-acquired pneumonia before the coronavirus disease 2019 (COVID-19) pandemic, especially in children aged 5–15 years old ^1, 2^. However, two large multicenter case-country studies, PERCH and GABRIEL, conducted between 2010 and 2014 in hospitalized children under 5 years of age with pneumonia, reported a detection rate of less than 2% ^3, 4^. High incidence rates tend to fluctuate in epidemiological waves, typically occurring every 3 to 7 years ^5– 7^. Only limited published data on *M. pneumoniae* infection in low and middle income country are available ^8– 11^. As far as is known *M. pneumoniae* only infects humans ^12– 14^. However, limited surveillance data hinder estimation of the true burden of *M. pneumoniae* infection. Similarities in presentation with other respiratory pathogens, and the absence of reliable point-of-care diagnostic tests, make it likely that the incidence of this infection is underestimated. Diagnosis is important as conventional treatment of respiratory tract infections with β-lactam antibiotics will not be efficacious for *M. pneumoniae*. *M. pneumoniae* has been described from adjoining Cambodia, Vietnam and Thailand, but not, as far as we aware, from Myanmar/Burma ^15, 16^. Little is known about the diversity of etiologies of acute respiratory infection (ARI) and incidence of *M. pneumoniae* in Laos. Currently prompt clinical diagnosis remains elusive as only one laboratory in Laos has demonstrated capability of testing for this pathogen. Few studies have been conducted, mainly on viruses ^17– 19^; *M. pneumoniae* has been reported in only one study, detected in 12 people with influenza-like illness from a community cohort study ^20^. Therefore there is a need for more information on the burden of M. pneumoniae infection in Laos. We describe here three patients positive for *M. pneumoniae*, to highlight the occurrence of this pathogen in Laos in children with ARI.

## Methods

From December 2013 to December 2014 we investigated the etiologies of ARI in hospitalized children at Mahosot Hospital, Vientiane ^21, 22^. This is an approximately 400-bed hospital providing primary, secondary, and tertiary care and receiving ≈2,000 inpatients/month.

Children aged <15-years-old admitted to paediatric wards with a clinical presentation compatible with ARI were included as previously described ^21^. Demographic, medical history, clinical and environmental data were collected by research physicians using a questionnaire, by interviews, physical examination and consulting medical charts ^22^. 

Written informed consent from the legal guardians of all patients was obtained before recruitment to the study. Ethical clearance was granted by the National Ethics Committee for Health Research, Ministry of Health, Vientiane, Laos, and the Oxford Tropical Research Ethics Committee (Oxford, UK).

Nasal and throat swab specimens were collected from included patients using Sigma Virocult® (Medical Wire & Equipment), then 100 µL of each were pooled, extracted and tested by real-time polymerase chain reaction (RT-PCR) for 33 pathogens using the FTD® respiratory pathogens 33 kit (FTD33, Fast-track Diagnostics), as previously described ^21^. A PCR assay was considered as positive if the Cq value was <35. FTD33 targets the Adhesin P1 gene of *M. pneumoniae* and validation by the manufacturer showed a detection limit of 100 copies/mL of plasmid and no reaction with *M. pneumoniae*-negative samples by real-time PCR reference methods (communication from Fast-track Diagnostics). No cross-reaction was observed when clinical samples containing 66 different bacterial, viral or parasitic pathogens were tested with FTD33 (communication from Fast-track Diagnostics). 

## Results

From December 2013 to December 2014, of 472 patients eligible and consenting to the study, 461 hospitalized with an ARI presentation were included (11 patients had missing clinical or laboratory data). One or multiple potential pathogens were detected in respiratory specimens of 447/461 (97.0%) patients ^22^ *M. pneumoniae* was detected in three patients, two girls and one boy, 5.7, 3.9 and 13.6 years old, respectively ( Table 1). All lived in Vientiane City and all had fever and cough. One patient had low haemoglobin (4.2g/dL), low haematocrit (13.3%) and high platelet count (8.43×10 ^5^/mm ^3^) on presentation, without other respiratory or gastroenteric signs or symptoms. The two other patients presented with runny nose, sore throat, and vomiting, in addition to cough. One patient had abnormal pulmonary auscultation on presentation and was hypoxic (O _2_ saturation of 90% on room air). None of these three patients had chest radiography performed.

**Table 1.** Characteristics of the three patients found PCR positive for *Mycoplasma pneumoniae* in the upper respiratory tract. All three patients were of Lao Loum ethnicity, all had documented fever and cough at enrolment, but none reported difficulty breathing, diarrhoea, chest indrawing, rash, cyanosis, respiratory distress, wheeze, stridor, nasal flaring, grunting, convulsion, conjunctival suffusion, lymphadenopathy, inability to drink, prostration or lethargy. None had known comorbidities at the time of presentation, or clinical evidence of pneumonia (WHO criteria ^23^), none required ICU admission and all were discharged alive with full recovery after two weeks. All symptoms for the three patients (rigors, sputum, runny nose, sore throat, vomiting, nausea) are consistent with *M. pneumoniae* infection.

	*M. pneumoniae* patients		
Patient	#1	#2	#3
Admission date	Feb 2014	Feb 2014	Sept 2014
Age (years)	5.7	13.6	3.9
Gender	female	male	female
Birth weight (g)	2,500	2,800	2,800
Ward	General Paediatrics	Infectious Disease Paediatric	Infectious Disease Paediatric
***Clinical presentation***			
Duration of illness prior to hospitalization (days)	2	6	4
Rigors	no	no	**yes**
Sputum	no	**yes**	no
Runny nose	no	**yes**	**yes**
Sore throat	no	**yes**	**yes**
Vomiting	no	**yes**	**yes**
Nausea	unknown	**yes**	no
***Physical examination***			
Abnormal pulmonary auscultation	no	**yes**	no
Oxygen saturation in room air (%)	99	90	unknown
Respiratory rate (breaths/min)	33	28	25
***Duration of hospitalisation *** ***(days)***	4	5	4
***Other organisms detected *** ***by*** FTD33			
Influenza B virus	**yes**	no	**yes**
Human cytomegalovirus	no	no	**yes**
*Staphylococcus aureus*	no	no	**yes**
*Streptococcus pneumoniae*	**yes**	no	**yes**
*Moraxella catarrhalis*	**yes**	no	**yes**
**CBC Test of periperhal ** **blood**			
White blood cells (10 ^3^ cells/mm ^3^)	7.10	3.32	8.92
Lymphocytes (10 ^3^ cells/mm ^3^)	1.04	1.55	1.98
Monocytes (10 ^3^ cells/mm ^3^)	0.50	0.18	0.26
Granulocytes (10 ^3^ cells/mm ^3^)	5.55	1.59	6.67
Lymphocytes (%)	14.7	46.8	22.2
Monocytes (%)	7.1	5.3	3
Granulocytes (%)	78.2	47.9	74.8
Hemoglobin (g/dL)	4.2	14.2	9.9
Mean corpuscular volume (fL)	59	83	79
Platelet count (10 ^3^ cells/mm ^3^)	843	112	151
**Treatment**			
	Paracetamol 250mg pro re nata	Paracetamol 500 mg pro re nata	Paracetamol 120mg x4 times/day
	Folic acid 5mg 1 time per day for 30 days	IV fluid	IV fluid
	Blood transfusion 2 times during admission (21 & 24 Feb 2014)	Vitamin C 500mg x3 times/day	Cefixime 50mg x 2 times/day, 1 day in hospital and continue at home
		Penicillin G 400,000 UI x 3 times/day for 5 days in hospital and continue for 5 days at home	

For the patient with abnormal pulmonary auscultation, *M. pneumoniae* was the only one of the 33 potential pathogens detected in the upper respiratory tract. Influenza B virus, *Streptococcus pneumoniae* and *Moraxella catarrhalis* were also detected in the two other patients. In addition, human cytomegalovirus and *Staphylococcus aureus* were also detected from one patient. Serology, culture and cold-aggultination assays were not available.

All patients received paracetamol to reduce fever. The patient with low haematocrit received a two-unit blood transfusion, whereas the two other patients received penicillin G and cefixime, respectively, during hospitalization. None received a macrolide, tetracycline or fluoroquinolone. After 4–5 days of hospitalization, all three patients improved and were discharged and 2 weeks later the three patients had fully recovered.

## Discussion

We describe three Lao children with *M. pneumoniae* infection, a pathogen that was first detected in Laos in 2019 ^20^. In the USA, the proportion of community-acquired pneumonia caused by *M. pneumoniae* increased following the introduction of pneumococcal vaccination ^12– 14^. The roll-out of pneumococcal vaccination in Laos since 2013 ^24^ therefore suggests that *M. pneumoniae* is a respiratory risk that warrants attention. However, there has been a very striking reduction in *M. pneumoniae* incidence in many countries during the COVID pandemic until recent resurgence in 2023 ^25, 26^.

Our study has important limitations, including that patients were recruited over only one year, and do not include recent data, and that we only collected upper respiratory tract samples. A pooled sample of nasal and throat swabs was used for *M. pneumoniae* detection by PCR, rather than the commonly accepted nasopharyngeal swab. However, we believe this did not significantly impact our results, as a recent study found higher sensitivity for *M. pneumoniae* detection in throat swabs compared to nasopharyngeal samples ^27^. No serology was performed and no published independent evaluation of the accuracy of the commonly used diagnostic kit used is available. The detection test used was PCR which is highly specific and sensitive; *M. pneumoniae* isolates of patient samples were not handled in the PCR laboratory, and therefore it is very unlikely that those results could be false positives due to contamination. While good laboratory practices were followed, contamination cannot be 100% ruled out. Results are likely to correspond to the co-detection of several micro-organisms (co-infections and/or carriage) in the upper respiratory tract as has been reported in other studies: more than one microorganism was detected in 73% of patients with ARI in the study in Laos by Phommasone *et al*. 2022 ^28^ and in 93% of cases (hospitalized children with severe pneumonia) in multiple countries in the PERCH study ^8^. All children improved without *M. pneumoniae* directed therapies, suggesting the infection may have been self-limiting. However, the possibility remains that *M. pneumoniae* was not the primary cause of illness in these cases.

Attributing causality of *M. pneumoniae* infection to disease is complicated, as with so many other respiratory pathogens, as a result of its occurrence in asymptomatic people. The three patients described here all had illnesses consistent with the described clinical features of *M. pneumoniae* ^12, 14^, but two were also PCR-positive for other potential respirarory pathogens. In addition, one of the girls had significant anaemia, raising the possibility of *M. pneumoniae* cold-agglutinin associated haemolytic anaemia, although other potential causes of anaemia were not investigated and cannot be excluded ^14^.

Further investigation of the importance of *M. pneumoniae* infection in Laos, and elsewhere where diagnostic services need reinforcing, is important for three key reasons. First, only one hospital in Laos has diagnostic facilities for *M. pneumoniae*, and these are not generally available for routine diagnosis in children with respiratory infection. Second, lacking a cell wall, *M. pneumoniae* is not susceptible to β-lactam antibiotics that would most commonly be used for respiratory tract infections ^23^. Macrolides, tetracyclines and fluoroquinolones are most commonly used to treat *M. pneumoniae* infection but with a poor evidence-base for efficacy ^11, 12^. More information is needed to understand which patients should receive empirical macrolide therapy in addition to β-lactam antibiotics. The revised 2020 Lao Pediatric Antimicrobial Prescribing Guidelines recommend oral amoxicillin and intravenous ampicillin as first line therapy for pneumonia not needing and requiring hospital admission, respectively, with option for oral switch to azithromycin, erythromycin or clarithromycin ^29^. When to add these anti- *Mycoplasma* antibiotics is difficult to judge given the paucity of local data and the fact that all three children described here recovered without receiving such agents. Third, the percentage of *M. pneumoniae* infection with macrolide-resistant *M. pneumoniae* is increasing worldwide, probably owing to the widespread use of this antibiotic class. Resistance frequencies in Asia are rising with reports that they represent the majority of isolates in some countries ^13, 30^. The resistance rate continues to be high following the COVID-19 pandemic ^31^. There are no data on the antibiotic resistance of *M. pneumoniae* from Laos in the public domain ^32^.

This study shows that *M. pneumoniae* existed in Laos in 2013–2014, without clarity on how often it causes infection. Surveillance research into the local prevalence of disease and antibiotic resistence patterns would assist in better understanding the epidemiology of *M. pneumoniae* infection and to inform treatment guidelines. More research on the current burden of *M. pneumoniae* in Laos is important in light of resurgence of *M. pneumoniae* infections post COVID-19 and recent data indicating high incidence of *M. pneumoniae* infection in young children in China in 2023 ^33^.

## Data Availability

All data underlying the results are available as part of the article and no additional source data are required.
