# Supplementary material for: Detection of Mycoplasma pneumoniae in hospitalized children with pneumonia in Laos
Source: Wellcome Open Res. 2025 Aug 7;8:533. Originally published 2023 Nov 20. [Version 5] doi: 10.12688/wellcomeopenres.19894.5 (PMC12759269; doi:10.12688/wellcomeopenres.19894.5)
Supplement: Peer review history

# Reviewer report: Reviewer response for version 5

**DOI:** 10.21956/wellcomeopenres.27284.r128666

**Published:** 2026-02-04

**Authors:** Daniel E Park

**Affiliations:** 1 George Washington University, Washington, USA

The authors have adequately addressed my primary concerns.

Is the work clearly and accurately presented and does it cite the current literature?

Partly

If applicable, is the statistical analysis and its interpretation appropriate?

Partly

Are all the source data underlying the results available to ensure full reproducibility?

Yes

Is the study design appropriate and is the work technically sound?

Yes

Are the conclusions drawn adequately supported by the results?

Partly

Are sufficient details of methods and analysis provided to allow replication by others?

Yes

Reviewer Expertise:

Epidemiology

I confirm that I have read this submission and believe that I have an appropriate level of expertise to confirm that it is of an acceptable scientific standard.

# Reviewer report: Reviewer response for version 5

**DOI:** 10.21956/wellcomeopenres.27284.r140557

**Published:** 2026-01-29

**Authors:** Anne Blaschke

**Affiliations:** 1 The University of Utah, Salt Lake City, Utah, USA

This paper describes the detection of *M. pneumoniae* in 3 children hospitalized in Laos in 2013-2014. While the authors are correct that the epidemiology of *M. pneumoniae* in Laos is understudied, this paper is a description of 3 patients in whom *M. pneumoniae* was detected, and not a study of the epidemiology of this pathogen. It is of interest that there were only 3 patients with *M. pneumoniae* detected – is this primarily due to limited availability of diagnostic testing? How does this rate of detection compare to rates in other LMIC? This is alluded to in the introduction but would be an important part of the analysis and discussion. The discussion has a very long paragraph about the importance of understanding antimicrobial treatment and resistance in *M. pneumoniae*, but this is not really related to the case series as there is no evidence that these patients had resistant organisms and they all got better without targeted therapy.

- The data analyzed are quite old (patients from 2013-2014) and even at the time of first submission the patients had been hospitalized up to 10 years prior. Given the worldwide changes in respiratory pathogen epidemiology since that time, it is not clear how relevant this paper is at this time.
- All 3 patients recovered without specific mycoplasma treatment. This suggests against a need to investigate resistance testing for this pathogen. For this manuscript, the discussion of resistance could be much shorter, and perhaps replaced with a discussion of the epidemiology in this study and comparison to others (even if they are limited).

Is the work clearly and accurately presented and does it cite the current literature?

Partly

If applicable, is the statistical analysis and its interpretation appropriate?

Not applicable

Are all the source data underlying the results available to ensure full reproducibility?

Yes

Is the study design appropriate and is the work technically sound?

Partly

Are the conclusions drawn adequately supported by the results?

No

Are sufficient details of methods and analysis provided to allow replication by others?

Yes

Reviewer Expertise:

I am a practicing physician with expertise in pediatric infectious diseases and a focus on the molecular epidemiolog of respiratory pathogens and diagnostics.

I confirm that I have read this submission and believe that I have an appropriate level of expertise to confirm that it is of an acceptable scientific standard, however I have significant reservations, as outlined above.

# Reviewer report: Reviewer response for version 5

**DOI:** 10.21956/wellcomeopenres.27284.r139977

**Published:** 2026-01-02

**Authors:** Holly B Schuh

**Affiliations:** 1 Johns Hopkins Bloomberg School of Public Health, Baltimore, USA

Investigators report clinical characteristics of three pediatric ARI cases with M. pneumoniae detected in the upper respiratory tract using data collected from 461 children at one hospital in Laos. Their discussion reviews the limited literature on the epidemiology of M. pneumoniae, the current clinical guidelines for treatment and their potential pitfalls, and the potential importance of further investigating M. pneumoniae as the etiological cause of ARI in Laos and the broader Asian region.

 Could the coauthors better clarify sampling by providing the number of screened, eligible, and consented participants to give readers a clear sense of representativeness at this hospital site?

 Since the data are fairly old by now, could the coauthors provide any further context on ARI epidemiology in Laos over the last five years? The Discussion section starts by stating that M. pneumoniae infection was first detected in Laos in 2019, which is 6-7 years after their data were collected. They go on to describe the introduction of the pneumococcal vaccination and the COVID-19 pandemic as potential influencers of M. pneumoniae infection. Are there any data/statistics/further published works that would provide better context on the influencers of M. pneumoniae infection, specifically in Laos? Any current data out of Laos? Without more information, I think that it is safer to say that the evidence provided in the current manuscript *may* warrant further investigation "of the importance of M. pneumoniae infection in Laos" (less definitive tone) in the second-to-last paragraph of the Discussion.

Is the work clearly and accurately presented and does it cite the current literature?

Yes

If applicable, is the statistical analysis and its interpretation appropriate?

Not applicable

Are all the source data underlying the results available to ensure full reproducibility?

No source data required

Is the study design appropriate and is the work technically sound?

Yes

Are the conclusions drawn adequately supported by the results?

Yes

Are sufficient details of methods and analysis provided to allow replication by others?

No

Reviewer Expertise:

infectious disease epidemiology, large-scale observational study designs, acute respiratory infections in children, clinical epidemiology

I confirm that I have read this submission and believe that I have an appropriate level of expertise to confirm that it is of an acceptable scientific standard, however I have significant reservations, as outlined above.

# Reviewer report: Reviewer response for version 4

**DOI:** 10.21956/wellcomeopenres.26733.r123375

**Published:** 2025-07-18

**Authors:** Nirma Vadlamudi

**Affiliations:** 1 Pharmaceutical Outcomes and Policy, The University of British Columbia, Gainesville, Florida, USA

This paper is a useful first step in documenting the presence of *M. pneumoniae* in Lao children with ARI, but its impact is limited by the very small number of cases, potential confounding by co-infections, and lack of comprehensive diagnostics. The study’s main value is in highlighting the need for larger, more current surveillance studies and improved diagnostic capacity in Laos to inform clinical management and public health policy.

 The authors should provide detail on other non- *M. pneumoniae* isolates and their clinical findings.

 Two of the three positive cases also had other respiratory pathogens detected. This raises questions about whether *M. pneumoniae* was the primary cause of illness or an incidental finding.

 The data are from 2013–2014, and the authors acknowledge that epidemiology may have changed, especially post-COVID-19. This reduces the current relevance of the findings.

 Authors should describe why a *M. pneumoniae *patient did not receive antibiotics.

 Overall, this paper should be combined with Dubot-Pérès *et al.* unpublished.

Is the work clearly and accurately presented and does it cite the current literature?

Yes

If applicable, is the statistical analysis and its interpretation appropriate?

Not applicable

Are all the source data underlying the results available to ensure full reproducibility?

Partly

Is the study design appropriate and is the work technically sound?

Partly

Are the conclusions drawn adequately supported by the results?

No

Are sufficient details of methods and analysis provided to allow replication by others?

Partly

Reviewer Expertise:

Epidemiology

I confirm that I have read this submission and believe that I have an appropriate level of expertise to confirm that it is of an acceptable scientific standard, however I have significant reservations, as outlined above.

# Response

**Published:** 2025-07-31

**Authors:** Audrey Dubot-Pérès

**Affiliations:** LOMWRU, University of Oxford, Vientiane, Lao People's Democratic Republic

**Reviewer comment: **The authors should provide detail on other non- *M. pneumoniae* isolates and their clinical findings.

**Response: **We appreciate the reviewer’s suggestion. The details regarding non- *M. pneumoniae* isolates and their associated clinical findings have already been published in two other publications, the references for which are provided in our revised version. (Nguyen et al. 2017, Dubot-Pérès et al., 2025).

**Reviewer comment: **Two of the three positive cases also had other respiratory pathogens detected. This raises questions about whether *M. pneumoniae* was the primary cause of illness or an incidental finding.

**Response: **We agree that the detection of co-infecting respiratory pathogens raises the possibility that *M. pneumoniae* may have been an incidental finding rather than the primary cause of illness. We have addressed this point in the manuscript, stating: “Attributing causality of *M. pneumoniae* infection to disease is complicated, as with so many other respiratory pathogens, due to its occurrence in asymptomatic individuals. The three patients described here all had illnesses consistent with the clinical features of *M. pneumoniae* [7,9], but two were also PCR-positive for other potential respiratory pathogens.”

**Reviewer comment: **The data are from 2013–2014, and the authors acknowledge that epidemiology may have changed, especially post-COVID-19. This reduces the current relevance of the findings.

**Response: **We acknowledge that the epidemiology of *Mycoplasma pneumoniae* may have evolved since 2014, particularly in the post-COVID-19 context. However, the lack of other published data on this topic in Laos highlights the importance of our study. We believe that sharing these findings remains valuable, as it is crucial to provide data on the circulation of *M. pneumoniae* in Laos.

**Reviewer comment: **Authors should describe why a *M. pneumoniae *patient did not receive antibiotics.

**Response: **We regret that we are unable to answer this as these decisions were made by the ward doctors and not by the study team. Moreover, the reason for not prescribing antibiotics was not captured in our study questionnaire, and we therefore do not have access to this information.

**Reviewer comment: **Overall, this paper should be combined with Dubot-Pérès *et al.* unpublished

**Response: **We would like to clarify that the manuscript by Dubot-Pérès et al. has already been published. That study focused specifically on the co-detection of *Streptococcus pneumoniae* and *Haemophilus influenzae *with RSV and influenza viruses and did not include any discussion of *Mycoplasma pneumoniae*. Including M. pneumoniae in that context would not have aligned with the scope or objectives of the publication and would have appeared out of place. For this reason, we believe it is appropriate to present the M. pneumoniae findings in a separate manuscript.

# Reviewer report: Reviewer response for version 4

**DOI:** 10.21956/wellcomeopenres.26733.r126810

**Published:** 2025-07-09

**Authors:** Daniel E Park

**Affiliations:** 1 George Washington University, Washington, USA

The authors have done a commendable job of addressing the peer reviews to date. I support that the information presented in the manuscript has limitations, but would be a helpful contribution to the literature as the region has a dearth of data on the subject matter. However, I believe given the substantial limitations, this should be written more similarly to a case report and thus have fewer population level conclusions. I have some minor comments for consideration and some general comments.

 General comments:

 - It would be important to contextualize findings with the understanding that Mycoplasma pneumoniae is known to have longer periodicity (3-7 years), with the study possibly occurring during a quiescent period.

 - The authors should consider whether portions of the discussion are directly linked with the findings of the study, which is essentially a case report of 3 children. More directly, it would not be prudent to inform population level perceptions of M. pneumoniae diagnosis, treatment, and antimicrobial susceptibility based on 3 patients. Thus, some of the information could either be omitted or shortened.

 - Results: The authors may want to consider whether a supplemental table comparing demographic and clinical characteristics of patients with and without M. pneumoniae. This is not essential as the n=3 is quite small, but it could give some indication as to the representativeness and characteristics of the source/total population.

 Minor comments:

 - Introduction: The estimate of 20-40% is both within epidemic periods and among specific age groups. Large standardized etiology studies such as PERCH, GABRIEL, and EPIC have demonstrated that the attributable fraction is much lower across children, likely in the <5% range. The nuances are important when framing the expected fraction in the study.

 - Introduction: While the revision to note the one hospital is helpful, it is now repeated in consecutive sentences towards the end of the first paragraph and the first sentence of the second paragraph. This construction seems unnecessarily repetitive.

 - Methods: What is the reference to the unpublished study at the end of the first sentence? The contents of the sentence do not seem to require a citation beyond the existing reference (#16).

 - Methods, paragraph 4: The authors should confirm that these are indeed nasal swabs. Nasal swabs potentially have lower sensitivity for M. pneumoniae detection compared with nasopharyngeal swab.

 - Results, Table 1: Adding the patient ID appears to not add any useful information to the table while also risking patient confidentiality.

 - Discussion, paragraph 1: As a stylistic choice, the first paragraph in the discussion typically summarizes the primary findings of the study. Including the false positive discussion here appears more of a response to reviewer questions rather than the main findings, and that line may be better suited for the limitations or later in the discussion.

 - Discussion, paragraph 1: The paragraph concludes that M. pneumoniae incidence was greatly reduced post-COVID. However, this is untrue, and is even contradicted by the concluding paragraph in the paper which points out the large outbreaks in China in 2023.

 - Discussion, paragraph 2: Many M *. pneumoniae* infections are self-limiting without *M. pneumoniae* directed therapies. This does not particularly address the question of whether *M*. *pneumoniae* was the primary etiologic cause or not. Note, pneumoniae is also mis-spelled in this sentence.

 - Discussion, paragraph 4: I believe “cell well” is a typo and should be “cell wall”

Is the work clearly and accurately presented and does it cite the current literature?

Partly

If applicable, is the statistical analysis and its interpretation appropriate?

Partly

Are all the source data underlying the results available to ensure full reproducibility?

Yes

Is the study design appropriate and is the work technically sound?

Yes

Are the conclusions drawn adequately supported by the results?

Partly

Are sufficient details of methods and analysis provided to allow replication by others?

Yes

Reviewer Expertise:

NA

I confirm that I have read this submission and believe that I have an appropriate level of expertise to state that I do not consider it to be of an acceptable scientific standard, for reasons outlined above.

# Response

**Published:** 2025-07-31

**Authors:** Audrey Dubot-Pérès

**Affiliations:** LOMWRU, University of Oxford, Vientiane, Lao People's Democratic Republic

We have prepared a new version of the manuscript taking into account the reviewers' comments.

**General comments:**

 -We have modified the macuscript to contextualize the findings by adding information about the periodicity in the introduction: “High incidence rates tend to fluctuate in epidemiological waves, typically occurring every 3 to 7 years (Yamazaki et al. 2016; Brown et al. 2016 ; Merișescu et al. 2025)”.

 -We have removed the portions of the discussion which are not directly linked with our findings.

 -Given the low number of *M. pneumoniae* patients, a comparison of demographic and clinical characteristics between patients with and without *M. pneumoniae* would not be much informative. In addition, the characteristics of the study patients are included in the published article Nguyen et al. 2017. Therefore, we don’t think it is necessary to add that information in supplemental table.

**Minor comments:**

 -We included to the introduction the *Mycoplasma pneumoniae* detection rates from the the PERCH and GABRIEL studies. We did not cite the EPIC study as its detection rate of 19% in children over 5 year old aligns with the previously mentioned estimate of 20-40%.

 - We have modified the end of the first paragraph and the beginning of the second paragraph to avoid the repetition.

 -The unpublished study we were refereing to has now been published, so we have added the corresponding reference.

 - As described in the method section, both nasal and throat swabs (not nasopharyngeal swab) were collected and then pooled. A recent study showed that sensitivity of *M. pneumonoiae* detection was significantly higher in oropharyngeal samples than in nasopharyngeal samples. However, this is probably not widely acknowledged, therefore we have added the sentence below to the limitations paragraph in the discussion section. "A pooled sample of nasal and throat swabs was used for M. pneumoniae detection by PCR, rather than the commonly accepted nasopharyngeal swab. However, we believe this did not significantly impact our results, as a recent study found higher sensitivity for *M. pneumoniae* detection in throat swabs compared to nasopharyngeal samples (Kitagawa et al. 2025)." -We have removed the patient ID from Table 1.

 -We have moved the sentence about false positive discussion to the paragraph on limitations. -We have modified the sentence to: “However, there has been a very striking reduction in M. pneumoniae incidence in many countries during the COVID pandemic until recent resurgence in 2023” .

 - We have modified the sentence as below: “All children improved without *M. pneumoniae* directed therapies, suggesting the infection may have been self-limiting. However, the possibility remains that *M. pneumoniae* was not the primary cause of illness in these cases”.

 -We have corrected the typo “cell wall”

# Reviewer report: Reviewer response for version 4

**DOI:** 10.21956/wellcomeopenres.26733.r123233

**Published:** 2025-06-13

**Authors:** Kristina Krohn

**Affiliations:** 1 Department of Internal Medicine and Department of Pediatrics, University of Minnesota Twin Cities, Minneapolis, Minnesota, USA

The manuscript is okay to be indexed. I have no further points to add.

Is the work clearly and accurately presented and does it cite the current literature?

Yes

If applicable, is the statistical analysis and its interpretation appropriate?

Yes

Are all the source data underlying the results available to ensure full reproducibility?

Yes

Is the study design appropriate and is the work technically sound?

Yes

Are the conclusions drawn adequately supported by the results?

Yes

Are sufficient details of methods and analysis provided to allow replication by others?

Yes

Reviewer Expertise:

NA

I confirm that I have read this submission and believe that I have an appropriate level of expertise to confirm that it is of an acceptable scientific standard.

# Reviewer report: Reviewer response for version 3

**DOI:** 10.21956/wellcomeopenres.26279.r120203

**Published:** 2025-03-25

**Authors:** Kristina Krohn

**Affiliations:** 1 Department of Internal Medicine and Department of Pediatrics, University of Minnesota Twin Cities, Minneapolis, Minnesota, USA

Thank you for your thorough response to my previous review. You have responded to all of my prior comments. My comments regarding the introduction and rephrasing or providing citations have been addressed by the changes in removing the Asia prevalence. This makes my related comment about the dating of that paper moot. 

**Introduction:**

 I appreciated your description in your response of the knowledge about *Mycoplasma pneumonia* in Laos. You stated, "There is no citation for this information, it comes from healthcare professional working in Laos and the fact that only one lab in the whole country can and has tested for this pathogen and it has only been described in Lao once before." I would suggest that rather than stating one person's opinion about *Mycoplasma pneumonia *knowledge as is done with the phrase "of which there is little apparent awareness in Laos and adjoining countries", it would be more accurate, and impactful to state the known facts that demonstrate that even if providers know that *M. pneumonia *exists in theory, they are unable to identify it in clinical practice as there is only one lab in the country that has demonstrated capability to identify this pathogen. 

 Consider rephrasing to something along the lines of "Diagnosis is important as conventional treatment of respiratory tract infections with β-lactam antibiotics will not be efficacious for *M. pneumoniae*. Currently prompt clinical diagnosis remains elusive as only one laboratory in Laos has demonstrated capability of testing for this pathogen."

**Conclusion:**

 - Similarly, the sentence about "awareness" of the diagnosis is an opinion statement. Providing a factual sentence on testing capabilities would be more compelling. You provide that later in the discussion, so I would delete this sentence. 

 - Thank you for moving away from the prior overstatements in the conclusion regarding that this study shows the need for studying antibiotic resistance in *Mycoplasma pneumoniae *infections. As currently stated, I am uncertain of the significance of resistance for *Mycoplasma pneumoniae *for Laos. I think the discussion of regional outbreaks and cases with resistance in China recently is more compelling on why it is important to include resistance patterns when working to add surveillance for *Mycoplasma pneumonia *in Laos.

Is the work clearly and accurately presented and does it cite the current literature?

Yes

If applicable, is the statistical analysis and its interpretation appropriate?

Yes

Are all the source data underlying the results available to ensure full reproducibility?

Yes

Is the study design appropriate and is the work technically sound?

Yes

Are the conclusions drawn adequately supported by the results?

Yes

Are sufficient details of methods and analysis provided to allow replication by others?

Yes

Reviewer Expertise:

NA

I confirm that I have read this submission and believe that I have an appropriate level of expertise to confirm that it is of an acceptable scientific standard, however I have significant reservations, as outlined above.

# Response

**Published:** 2025-05-02

**Authors:** Audrey Dubot-Pérès

**Affiliations:** LOMWRU, University of Oxford, Vientiane, Lao People's Democratic Republic

Introduction: Thank you for the suggestion, we have modify the text as suggested.   Conclusion: Thank you for the suggestion, we have removed the sentence about “awarenss”. We are already mention the resecent increase in resistance in Asia and cite the recent review “New Insights into the Epidemiological Characteristics of Mycoplasma pneumoniae Infection before and after the COVID-19 Pandemic”. Therefore we are not sure if there is a specific request from the reviewer.

# Reviewer report: Reviewer response for version 2

**DOI:** 10.21956/wellcomeopenres.24901.r115279

**Published:** 2025-01-16

**Authors:** Yan Chen

**Affiliations:** 1 Zhejiang University School of Medicine, Hangzhou, Zhejiang, China

This study addresses an important topic by investigating the role of *Mycoplasma pneumoniae* in community-acquired pneumonia among pediatric inpatients in Laos. The use of PCR for pathogen detection is a strength, highlighting the study’s methodological foundation. However, the manuscript has significant limitations that undermine its impact and reliability. The extremely low positivity rate for *M. pneumoniae* raises concerns about the diagnostic accuracy or methodological limitations, while the analysis and interpretation of the results are insufficiently developed. The study does not provide robust evidence to support the conclusions drawn, limiting its contribution to understanding the epidemiology of *M. pneumoniae*
 

1. This study is a retrospective analysis of *Mycoplasma pneumoniae* detection and clinical information collection from pediatric inpatients at Mahosot Hospital between 2013 and 2014 using PCR. However, concerns arise regarding the accuracy of the data and results. While the study mentions 461 children as subjects, the focus on only three positive cases is not adequately explained. It is unclear whether the sample size is statistically representative. The low *M. pneumoniae* positivity rate compared to other reports raises questions about the reliability of the detection method used, yet the authors do not discuss this potential limitation.
2. The results section lacks sufficient detail, providing only clinical information about the patients. It does not describe the methods of data analysis or include comparisons or statistical discussions among case groups. Even with a small sample size, some preliminary statistical analysis should be provided. Furthermore, there is a disconnect between the results and the discussion sections, which undermines the cohesion of the study.
3. The conclusion suggests enhancing diagnostic systems and analyzing antibiotic resistance for *M. pneumoniae*. However, it fails to substantiate why these measures are necessary, especially considering the study reports an exceptionally low *M. pneumoniae* positivity rate in Laos. This discrepancy should be addressed and supported by the findings.

Is the work clearly and accurately presented and does it cite the current literature?

Partly

If applicable, is the statistical analysis and its interpretation appropriate?

Partly

Are all the source data underlying the results available to ensure full reproducibility?

Partly

Is the study design appropriate and is the work technically sound?

Partly

Are the conclusions drawn adequately supported by the results?

Partly

Are sufficient details of methods and analysis provided to allow replication by others?

No

Reviewer Expertise:

infectious diseases; virulence; resistance mechanism

I confirm that I have read this submission and believe that I have an appropriate level of expertise to state that I do not consider it to be of an acceptable scientific standard, for reasons outlined above.

# Response

**Published:** 2025-02-16

**Authors:** Audrey Dubot-Pérès

**Affiliations:** LOMWRU, University of Oxford, Vientiane, Lao People's Democratic Republic

We are unclear about the reviewer’s comment suggesting that our finding (low positive rate) is inconsistent with other reports. It is possible that in Laos, *M. pneumoniae* infection plays a smaller role in community-acquired pneumonia, or that the samples we collected were taken outside of epidemic periods. We utilized a real-time PCR assay, recognized as the gold standard for sensitive pathogen detection, using the FTD33 kit, which has been widely employed in numerous studies, including the PERCH study, which includes a control population. Only one study on *Mycoplasma pneumoniae* has been published in Laos, where the positive rate was higher than in our study (14/548). However, the study design differed, even though the same assay was used for detection.

 This is a descriptive study, and given that only 3 patients tested positive for *M. pneumoniae*, I’m unsure what reliable statistical analysis could have been performed. Moreover, the purpose of this paper is not to estimate the incidence of *M. pneumoniae* or analyze risk factors, but rather to highlight its presence in Laos and emphasize the need for increased surveillance and research to better understand its epidemiology and ensure the availability of appropriate diagnosis and treatment. The aim of this article is to demonstrate the presence of M. pneumoniae in order to raise awareness about the need for further research to better understand its epidemiology in Laos, as there has been no specific research conducted on this pathogen and no laboratories currently perform its diagnosis. Given the public health significance of this pathogen in other countries, it seems crucial to publish even limited findings in an effort to increase awareness and highlight the need for more research.

# Reviewer report: Reviewer response for version 2

**DOI:** 10.21956/wellcomeopenres.24901.r102223

**Published:** 2024-10-14

**Authors:** Chao Shi

**Affiliations:** 1 Qingdao University, Qingdao, Shandong, China

The authors mentioned in the abstract that is needed to raise awareness and improve understanding of the epidemiology of *M. pneumoniae* infection in Laos, enable targeted therapy. The authors highlighted the need for better diagnostic tools and the understanding of antibiotic resistance to improve treatment and management of *M. pneumoniae* infection in Laos.

 The authors investigated at Mahosot Hospital in Vientiane, Laos, examined 461 children with acute respiratory infections from December 2013 to December 2014. Using a PCR test, *M. pneumoniae* was found in the upper respiratory tracts of three children.

1. The positive results of *M. pneumoniae* detection by PCR only accounted for 0.6% of total number. In fact, this set of data could not explain the importance of diagnosis of *M. pneumoniae*. Besides, only the specificity of FTD33 kit has been verified, but have not been checked by other methods, such as serology. We suggest amending this.
2. And the results of this survey are inconsistent with introduction that *M. pneumoniae* infection, which occurs worldwide with both endemic and epidemic patterns, may have been responsible for up to 20–40% of community-acquired pneumonia before the coronavirus disease 2019 (COVID-19) pandemic.
3. This article aims to improve the treatment of *M. pneumoniae* infection in Laos by improving the understanding of the epidemiology of *M. pneumoniae* infection in Laos. Therefore, we suggest analyzing the importance of distinguishing *M. pneumoniae* infection from other respiratory infections with the same symptoms from the serious consequences such as drug-resistant infection caused by antibiotic abuse, and then clarify the significance of *M. pneumoniae* diagnosis.

Is the work clearly and accurately presented and does it cite the current literature?

Partly

If applicable, is the statistical analysis and its interpretation appropriate?

Not applicable

Are all the source data underlying the results available to ensure full reproducibility?

Yes

Is the study design appropriate and is the work technically sound?

Partly

Are the conclusions drawn adequately supported by the results?

No

Are sufficient details of methods and analysis provided to allow replication by others?

Partly

Reviewer Expertise:

Molecular biology；Nucleic acid testing；POCT；Biosensor

I confirm that I have read this submission and believe that I have an appropriate level of expertise to state that I do not consider it to be of an acceptable scientific standard, for reasons outlined above.

## References

[1] : Epidemiology, clinical manifestations, pathogenesis and laboratory detection of Mycoplasma pneumoniae infections. *FEMS Microbiol Rev* .2008;32(6) : 10.1111/j.1574-6976.2008.00129.x 956-73 10.1111/j.1574-6976.2008.00129.x 18754792

[2] : Clinical Evaluation of a Novel Point-of-Care Assay To Detect Mycoplasma pneumoniae and Associated Macrolide-Resistant Mutations. *J Clin Microbiol* .2021;59(7) : 10.1128/JCM.03245-20 e0324520 10.1128/JCM.03245-20 33910960 PMC8218770

[3] : Detection of Mycoplasma pneumoniae in Mexican children with community-acquired pneumonia: experience in a tertiary care hospital. *Infect Drug Resist* .2019;12: 10.2147/IDR.S193076 925-935 10.2147/IDR.S193076 31118700 PMC6503500

# Response

**Published:** 2025-02-16

**Authors:** Audrey Dubot-Pérès

**Affiliations:** LOMWRU, University of Oxford, Vientiane, Lao People's Democratic Republic

We agree that our data cannot explain the importance of *M. pneumoniae *in Laos, and this is not what we state, we just report the detection of *M. pneumoniae* which as been described in Laos only once before. We clearly said in the discussion that “Further investigation of the importance of *M. pneumoniae* infection in Laos is important”.

 We do not understand why this is inconsistent. Perhaps in Laos *M. pneumoniae* infection is responsible for less community-acquired pneumonia, or the sample collected were outside epidemics.

 No study has been conducted to estimate this, and this is why our manuscript is important to enhance awarness on the need for research on *M. pneumoniae* to understand the epidemiology and estimate the burden in Laos. Our motivation for writing this paper is not to over-, or underestimate, the incidence of *M. pneumoniae* but to point out that it is in Lao and that more surveillance and research is needed to better understand its epidemiology and ensure that appropriate diagnosis and treatment is available. The aim of the article is to enhance awarness on the need for more research to better understand the epidemiomogy of *M. pneumoniae* in Laos and to inform treatment guidelines (additionnal research that would be conducted in the future would permit to inform treatment guidelines not this current article).

# Reviewer report: Reviewer response for version 2

**DOI:** 10.21956/wellcomeopenres.24901.r88599

**Published:** 2024-08-15

**Authors:** Kristina Krohn

**Affiliations:** 1 Department of Internal Medicine and Department of Pediatrics, University of Minnesota Twin Cities, Minneapolis, Minnesota, USA

**Overall comments:**

- I will still defer to the editors on questions from my previous review.
- Again, I think noting the *M. pneumonia *was present in Laos is appropriate and worth indexing. The current version still overstates the findings. Toning this down to only stating what can be determined from this current study and citing anything that is from other information is important for accuracy.

**Abstract:**

- It looks like you have re-written the abstract but have left the prior abstract still there. Please delete the first paragraph of the abstract.
- “Other germs were also detected in the girls.” This is too colloquial for a scientific paper. Please re-write back to “The two girls were also positive for other potential pathogens.” 
- Because it is very important in your study that these children improved without *M pneumoniae* directed therapy, please add that to the abstract.
- Your study primarily suggests that clinicians should think of *M. pneumonia *as a potential pathogen and consider testing for it. Your study doesn’t actually look at resistance patterns, so while important, I would not include that in the abstract. Therefore, add the need to improve diagnosis and detection (not just improved tools) of *M. pneumonia* to the last line, as in “The study highlights the need for improving the diagnosis and understanding of *M. pneumoniae *infection in Laos.”

**Introduction:**

- This sentence “For reasons that are not understood, *M. pneumoniae* appears to be more common in Asia than elsewhere.” Should be referenced.
- Also, it may be relevant to state “ *M. pneumoniae* appeared to be more common in Asia than elsewhere before the COVID-19 pandemic” *if *you are unable to find more recent epidemiologic data.
- This statement “of which there is little awareness in Laos and adjoining countries.” Should be cited.
- The last line of the introduction overstates what can be accurately interpreted from this study.  Please consider rewording, such as “We describe here three patients positive for *M. pneumoniae* to highlight that this pathogen can occur in Laos.”

**Methods:**

- The parenthetical phrase that Reference 16 is unpublished contradicts the citation in the references
- Please rephrase to “This is an approximately 400 bed hospital…”
- Please confirm that the Dubot-Pérès cited at the end of the second paragraph is indeed unpublished, or if it too is reference 16.

**Table 1:**

- The following information should not be in the table, but in the text/limitations section.
  “Contamination cannot be 100% ruled out, however the test were performed following good laboratory practice using quality controls. Those results are likely to correspond to co-detection of several micro-organism (co-infections and/or carriage) in upper respiratory tract as it is reported in other studies: more than one microorganism was detected in 73% of patients with ARI in the study in Lao by Phommasone et al. 2022 and in 93% of cases (hospitalized children with severe pneumonia) in the PERCH study.”

**Results:**

- 843 x 10 ^3^ is not accurate scientific notation. Please change to 8.43 x 10 ^5^.

**Discussion:**

- The information provided does not support the sentence “The detection test used was PCR which is highly specific and sensitive; *M. pneumoniae* isolates of patient samples were not handled in the PCR laboratory, and therefore it is very unlikely that those results could be false positives due to contamination.” There is not adequate information in this study to assess the specificity and sensitivity. Nor is the fact that the samples were done outside of the PCR lab make it less likely to be contaminated, even the very best labs sometimes have contamination. In any event, this sentence is unnecessary, and I’d suggest simply deleting it.
- The statement of little awareness would benefit from some sort of citation or explanation.
- Limitations paragraph. The best studies are very transparent about their limitations. Please expand on this section. The paragraph from the Table should be added here. 
  For example, “Our study has limitations, including patients collected over only one year, not including recent data, and only collecting upper respiratory tract samples. No serology was performed and no published evaluation of the accuracy of the diagnostic kit used is available. While good technique was utilized, contamination cannot be 100% ruled out. More than one microorganism was detected for two of the three children, which calls in to question which pathogen was causing disease. However, co-detection of *M. *pneumonia in 73% of patients with ARI in the study in Lao by Phommasone et al. 2022 and in 93% of cases (hospitalized children with severe pneumonia across Africa and Asia) in the PERCH study. All children improved without *M. pneumonia *directed therapies, which raises the question of if *M. pneumonia *was causing infection or if it was merely present.
-  A period is needed at the end of this sentence “The revised 2020 Lao Pediatric Antimicrobial Prescribing Guidelines recommend oral amoxicillin and intravenous ampicillin as first line therapy for pneumonia not needing and requiring hospital admission, respectively, with option for oral switch to azithromycin, erythromycin or clarithromycin ^23^ ” (note: recommend has 2 “m”s)
- The “First”, “Second”, and “Third” transitions are awkward in that the first two occur within a paragraph and the third is the start of a new paragraph. Consider adapting to by making the sentence “Further investigation of the importance of *M. pneumoniae* infection in Laos, and elsewhere where diagnostic services need reinforcing, is important for three key reasons” the start of its own paragraph. Then either include all 3 points in that paragraph or make them each their own bulleted paragraph underneath.
- “Third, macrolide-resistant *M. pneumoniae* has become increasingly prevalent worldwide, probably owing to the widespread use of this antibiotic class.” Is not a completely accurate statement because of the decrease in overall *M. pneumoniae *infections post COVID. Rephrasing to “Third, the percentage *M. pneumoniae *infections with macrolide-resistant *M. pneumoniae* is increasing worldwide, probably owing to the widespread use of this antibiotic class.”
- There should be a comment about how the COVID-19 pandemic affected resistance patterns if possible.
- The last paragraph over states the implications of this work. Please reframe. For example, “This study shows that *M. pneumoniae *existed in Laos in 2013-2014, without clarity on how often it causes infection. Surveillance research into the local prevalence of disease and antibiotic resistance patterns would assist in better understanding the epidemiology of *M. pneumoniae* infection and to inform treatment guidelines. Better understanding of the current burden of *M. pneumoniae* in Laos is important now in light of anticipated resurgence of *M. pneumoniae *infections post COVID-19 and recent data indicating a high incidence of M. pneumoniae infection in young children in China in 2023 ^27^ .”

Is the work clearly and accurately presented and does it cite the current literature?

Yes

If applicable, is the statistical analysis and its interpretation appropriate?

Yes

Are all the source data underlying the results available to ensure full reproducibility?

Yes

Is the study design appropriate and is the work technically sound?

Yes

Are the conclusions drawn adequately supported by the results?

Yes

Are sufficient details of methods and analysis provided to allow replication by others?

Yes

Reviewer Expertise:

NA

I confirm that I have read this submission and believe that I have an appropriate level of expertise to confirm that it is of an acceptable scientific standard, however I have significant reservations, as outlined above.

# Response

**Published:** 2025-02-16

**Authors:** Audrey Dubot-Pérès

**Affiliations:** LOMWRU, University of Oxford, Vientiane, Lao People's Democratic Republic

**Abstract:**

- We did not rewrite the abstract, the 2nd paragraph was not in the submitted manuscript, and am not sure where it is coming from.  Could the 2nd paragraph be deleted by the journal ?
- Agree, we will keep the 1st paragraph and therfore this sentence will be deleted.
-  Thank you, we have added this sentence to the abstract
- The 2nd paragraph must be removed, the 1st paragraph, which is the correct abstract, mentions the need for “enhancement of diagnostic systems for *M. pneumoniae* detection to improve understanding of the epidemiology of *M. pneumoniae* infection in Laos”. We have removed the mention of resistant profile as suggested.

**Introduction:**

- This is from an outdated paper and we have removed this statement that no longer holds true.
- We were unable to find data supporting this. We would appreciate it if the reviewer could provide the relevant reference.
-  This information is based on the experience of individuals from the region. We have revised the statement to: “of which there is little apparent awareness in Laos and adjoining countries”.
- Thank you, we have modified the sentence accordingly: “We describe here three patients positive for *M. pneumoniae*, to highlight the occurrence of this pathogen in Laos in children with ARI.”

**Introduction:**

-  The unpublished manuscript we are mentionning under brackets (Dubot-Pérès *et al*. unpublished) is different from the reference 16.
- The modification has been done.
- The unpublished Dubot-Pérès et al and the reference 16 are 2 different manuscripts.

**Table 1:**

 The following paragraph has been moved to the discussion in limitations section.

 “While good laboratory practices were followed, contamination cannot be 100% ruled out. Results are likely to correspond to the co-detection of several micro-organisms (co-infections and/or carriage) in the upper respiratory tract as has been reported in other studies: more than one microorganism was detected in 73% of patients with ARI in the study in Laos by Phommasone *et al.* 2022 ^18 ^and in 93% of cases (hospitalized children with severe pneumonia) in multiple countries in the PERCH study”.

**Results:** The modification has been done.

**Discussion:**

- I agree that contamination occurs even in laboratories with best practices, however the likelihood for contamination to occurr is related to the way the different lab activities and the workflow are organized. In our lab, microorganism culture is completely physically separated from the molecular lab, with different staff working in the different laboratories and *M. pneumoniae *isolates were not used for molecular testing. Henece, the risk for contamination is decreased compared to laboratories in which isolates are regularly extracted and submitted for molecular testing, with high concentrations of DNA handled in molecular laboratory. Therefore, I think this is important to mention here. As a molecular scientist, I find it reassuring to include  this information, reducing the suspiscion that this result could come from contamination from isolate DNA if handled in the same lab.
- As mentionned above, there is no citation for this information, it comes from helathcare professionnal working in Laos and the fact that only one lab in the whole country can and has tested for this pathogen and it has only been described in Lao once before.
- The limitations paragraph has been modified as suggested: “Our study has important limitations, including that patients were recruited over only one year, and do not include recent data, and that we only collected upper respiratory tract samples. No serology was performed and no published independent evaluation of the accuracy of the commonly used diagnostic kit used is available. While good laboratory practices were followed, contamination cannot be 100% ruled out. Results are likely to correspond to the co-detection of several micro-organism (co-infections and/or carriage) in the upper respiratory tract as has been reported in other studies: more than one microorganism was detected in 73% of patients with ARI in the study in Laos by Phommasone *et al.* 2022 ^18^ and in 93% of cases (hospitalized children with severe pneumonia) in multiple countries in the PERCH study ^3^. All children improved without *M. pneumonia *directed therapies, which raises the question of whether *M. pneumonia *was causing the disease”.
- Thank you, modification has been made.
-  Thank you for the suggestion, we have modified all three points in the same paragraph.
- We have modified as suggested.
- A review from wu *et al.* (2024)  reported that resistance remains high after COVID-19 pandemic. We added this to the manuscript. We added the following sentence “The resistance rate continues to be high following the COVID-19 pandemic”.
- We have modified the last paragraph as below: “This study shows that M. pneumoniae existed in Laos in 2013-2014, without clarity on how often it causes infection. Surveillance research into the local prevalence of disease and antibiotic resistence patterns would assist in better understanding the epidemiology of M. pneumoniae infection and to inform treatment guidelines. More research on the current burden of M. pneumoniae in Laos is important in light of resurgence of M. pneumoniae infections post COVID-19 and recent data indicating high incidence of M. pneumoniae infection in young children in China in 2023.”

# Reviewer report: Reviewer response for version 1

**DOI:** 10.21956/wellcomeopenres.22029.r72446

**Published:** 2024-01-27

**Authors:** Robert HEYDERMAN

**Affiliations:** 1 University College London, London, UK

The authors have investigated 461 children admitted to Mahosot Hospital, Vientiane, Laos, with acute respiratory infection defined by onset of symptoms <14 days, fever (axillary temperature >38.0 °C) or history of fever, and at least one respiratory symptom (dyspnoea, cough, rhinitis) or abnormal pulmonary auscultation on physical examination between December 2013 to December 2014. Pooled nasal and throat swabs were tested using a 33-pathogen multiplex PCR (FTD33). *Mycoplasma pneumoniae* infection was detected in three children 6, 4 years old and 14 years old. In one child, influenza B virus, *Streptococcus pneumoniae* and *Moraxella catarrhalis* were also detected. In another, influenza B virus, *Streptococcus pneumoniae*, CMV, *Staphylococcus aureus* and *Moraxella catarrhalis* were all detected.

1. No confirmatory serology was performed.
2. No radiology was available (frequently not done routinely in children with ARI).
3. All three children recovered without specific anti-mycoplasma antimicrobial therapy. One child had severe anemia, but cold agglutinins were not measured.
4. Therefore, although mycoplasma infection was likely present, mycoplasma-associated disease was not convincingly proven. Even if these cases do represent mycoplasma disease, they comprise only 0.6% of a cohort recruited 10 years ago.
5. The authors statement that these data support the enhancement of “diagnostic systems for *M. pneumoniae* detection and analysis of its antibiotic resistance profile to raise awareness and improve understanding of the epidemiology of *M. pneumoniae* infection in Laos, enable targeted therapy, and inform treatment guidelines” is not substantiated by their data.
6. Indeed, it would be hard to justify the diversion of public health or clinical research resources based on these findings. To better understand the burden of *M. pneumoniae-associated* respiratory disease, a more contemporary cohort of children fulfilling the criteria for community acquired pneumonia/ severe acute respiratory infection would have been more informative.
7. The authors have not provided a sufficiently authoritative review of the relevant literature from LMICs (I have provided some examples of studies that could have been included).
8. The authors make no mention of the potential for *M. pneumoniae *to cause epidemics in Asia. The impact of the COVID-19 pandemic on *M. pneumoniae* epidemiology should have been considered more carefully as a limitation to the relevance of this data from 2014.

Is the work clearly and accurately presented and does it cite the current literature?

Partly

If applicable, is the statistical analysis and its interpretation appropriate?

Not applicable

Are all the source data underlying the results available to ensure full reproducibility?

Yes

Is the study design appropriate and is the work technically sound?

Partly

Are the conclusions drawn adequately supported by the results?

No

Are sufficient details of methods and analysis provided to allow replication by others?

Partly

Reviewer Expertise:

My research bridges clinical practice, large-scale field studies, disease prevention and the fundamental understanding of the mechanisms of infectious disease.

I confirm that I have read this submission and believe that I have an appropriate level of expertise to state that I do not consider it to be of an acceptable scientific standard, for reasons outlined above.

## References

[1] : Causes of severe pneumonia requiring hospital admission in children without HIV infection from Africa and Asia: the PERCH multi-country case-control study. *Lancet* .2019;394(10200) : 10.1016/S0140-6736(19)30721-4 757-779 10.1016/S0140-6736(19)30721-4 31257127 PMC6727070

[2] : Aetiology of childhood pneumonia in a well vaccinated South African birth cohort: a nested case-control study of the Drakenstein Child Health Study. *Lancet Respir Med* .2016;4(6) : 10.1016/S2213-2600(16)00096-5 463-72 10.1016/S2213-2600(16)00096-5 27117547 PMC4989125

[3] : Epidemiology and Molecular Identification and Characterization of Mycoplasma pneumoniae, South Africa, 2012-2015. *Emerg Infect Dis* .2018;24(3) : 10.3201/eid2403.162052 506-513 10.3201/eid2403.162052 29460736 PMC5823326

[4] : Annual and seasonal patterns in etiologies of pediatric community-acquired pneumonia due to respiratory viruses and Mycoplasma pneumoniae requiring hospitalization in South Korea. *BMC Infect Dis* .2020;20(1) : 10.1186/s12879-020-4810-9 132 10.1186/s12879-020-4810-9 32050912 PMC7017508

# Response

**Published:** 2024-06-14

**Authors:** Audrey Dubot-Pérès

**Affiliations:** LOMWRU, University of Oxford, Vientiane, Lao People's Democratic Republic

Thank you for the comments.

 4 : However, very few data on *M. pneumoniae* in Laos are available, with the diagnostic test available in only one hospital in Laos. Even if there is not indisputable evidence that *M. pneumoniae* was the cause of the disease, our results are evidence that the organism is circulating in children in Laos. Therefore, we suggest that it is important to report that fact and draw attention on the need for more investigation to know the true burden in Laos, spread awareness and alertness to this disease and optimize the treatment of children with ARI.  

 5: Same comment as above. The organism is circulating, and therefore there is a need to investigate more in details, to develop appropriate diagnostic facilities, awareness and optimize the treatment of children with ARI. If subsequent research demonstrate that it is a very rare cause we would be delighted as would simplify treatment of ARI but at the moment such data do not exist, leaving a gap in ARI pathogen surveillance  

 6: Thank you for the suggestion and indeed this is what we are suggesting: more research investigation to understand the true burden of *M. pneumoniae* in Laos, especially in the view of the recent epidemics in China. We added the following sentence at the end of the discussion: “More research on the burden of *M. pneumoniae* in Laos is all the more crucial in light of recent data indicating high incidence of M. pneumoniae infection in young children in China in 2023 ^22^”.  

 7: We added more references to the sentence below in introduction: “Only limited published data on *M. pneumoniae* infection in low and middle income country are available ^3–6.^”  

 8: The recent *M. pneumoniae* epidemic in China shows the relevance of our paper, the aim of which is to draw attention to the importance of investigating M. pneumoniae infections in Laos and elsewhere in SE Asia.

# Reviewer report: Reviewer response for version 1

**DOI:** 10.21956/wellcomeopenres.22029.r70482

**Published:** 2023-11-30

**Authors:** Kristina Krohn

**Affiliations:** 1 Department of Internal Medicine and Department of Pediatrics, University of Minnesota Twin Cities, Minneapolis, Minnesota, USA

**Detection of *Mycoplasma pneumoniae* in hospitalized children with pneumonia in Laos [version 1; peer review: awaiting peer review]**

 Overall: I like this article. I think it is good to know that *Mycoplasma pneumoniae *is a potential pathogen in Laos. I have a few concerns, some of which are easily addressed. First, this article does need a limitations section. In particular there should be some discussion of the accuracy of the results attained and how reliable the PCR testing is in this setting. All tests have a specificity and sensitivity that should be shared. The positive and negative predictive values in this setting were population prevalence is utterly unknown is more difficult. Second, further characterization about what is consistent and what is inconsistent with *Mycoplasma pneumoniae *clinical presentations should be included. Lastly, I am uncertain and defer to the editors about how it appears the disease specific data appears to be presented as separate studies for an overarching acute respiratory infection study. 

 Abstract:  No comments

 Intro:

- Is *Mycoplasma pneumoniae* smaller than *Mycoplasma genitalium?  *I think that Mycoplasma as a whole are considered the smallest, but I think genitalium is smaller than pneumoniae. I would defer to others here.
- Several statements from the conclusion should be included in the intro to show to readers the importance of this article. Specifically the following:
  - *“M. pneumoniae* has been described from adjoining Cambodia, Vietnam and Thailand, but not, as far as we are aware, from Myanmar/Burma ^14, 15^. Further investigation of the importance of *M. pneumoniae* infection in Laos, and elsewhere where diagnostic services need reinforcing, is important for three key reasons. First, only one hospital in Laos has diagnostic facilities for *M. pneumoniae*, and these are not generally available for routine diagnosis in children with respiratory infection. Second, lacking a cell well, *M. pneumoniae* is not susceptible to β-lactam antibiotics that would most commonly be used for respiratory tract infections ^11^. Macrolides, tetracyclines and fluoroquinolones are most commonly used to treat *M. pneumoniae* infection but with a poor evidence-base for efficacy ^3, 4^. More information is needed to understand which patients should receive empirical macrolide therapy in addition to β-lactam antibiotics. The situation is analogous to the treatment of central nervous system infections in Laos and elsewhere in rural Asia, to cover not just the ‘conventional’ bacteria but also the common causes that are regarded wrongly as ‘atypical’, such as scrub typhus and murine typhus, that require non-β-lactam antibiotics for effective therapy ^16, 17^. The revised 2020 Lao Pediatric Antimicrobial Prescribing Guidelines recommend oral amoxicillin and intravenous ampicillin as first line therapy for pneumonia not needing and requiring hospital admission, respectively, with option for oral switch to azithromycin, erythromycin or clarithromycin ^18^ When to add these anti- *Mycoplasma* antibiotics is difficult to judge given the paucity of local data “

Methods:

- It appears that this is a subset of the data for from an overarching unpublished study in reference # 10.  It looks like the methods, etc. were similar but the previous published data is for RSV, while this is only looking at Mycoplasma. Ideally the complete study would have been published together, perhaps with editorials or perspective articles providing additional perspective on specific illnesses.

Results:

- It is unclear why the sex is shared in relation to the abnormal pulmonary auscultation and hypoxia. Either share the sex when it is relevant to the findings only, or don’t share it at all or share it all the time. If sharing only when it is relevant, then please explain why it is relevant.
- It is unclear why the 3.9-year-old female had so many positive organisms (similar for the 5.7-year-old). Are these false positives? If she did have all of these I would expect her to be very sick and immunocompromised, or at least to have an elevated WBC. It seems likely that there was an error in the test (contamination?) or the WBC was done after significant treatment?

Discussion:

- The primary argument of this paper – that *Mycoplasma pneumoniae *is an entity that should be considered in Laos is important. That there are positive tests at all is suggestive of this. Several factors should be further discussed to flush out this argument.
  - What are the reasons for false positive results or other reasons to question these results? I.e. lab contamination, or testing that has lower specificity?
  - How does the presentation of infection compare to that other places? I appreciate the comment that the anemia could be cold agglutination. I also appreciate the comment about all 3 improving without the recommended treatment. How often does that occur in other settings? Also, the ages… in many recommendations azithromycin is recommended after a given age (ex. Above 8-years-old).  How does this compare to ages of hospitalized infection from *Mycoplasma pneumoniae* in other places.
- *Mycoplasma pneumoniae*is thought to be an outpatient presentation. What percentage of infections are thought to cause hospitalizations? If there are 3 in this sample, what would the expected number of cases be that would present to outpatient care? Or be asymptomatic?
- What other factors should be included in a discussion of whether or not to change recommendations? Or is it simply enough for now to know that this is a possibility.

Is the work clearly and accurately presented and does it cite the current literature?

Yes

If applicable, is the statistical analysis and its interpretation appropriate?

Yes

Are all the source data underlying the results available to ensure full reproducibility?

Yes

Is the study design appropriate and is the work technically sound?

Yes

Are the conclusions drawn adequately supported by the results?

Yes

Are sufficient details of methods and analysis provided to allow replication by others?

Yes

Reviewer Expertise:

I study medical education and medical communication, including having taught clinical practice in Laos. I can comfortably identify that the information in this article is important to clinicians in Laos. When it comes to specifics laboratory research and pathogen identification, that is beyond my scope of practice.

I confirm that I have read this submission and believe that I have an appropriate level of expertise to confirm that it is of an acceptable scientific standard, however I have significant reservations, as outlined above.

# Response

**Published:** 2024-06-10

**Authors:** Audrey Dubot-Pérès

**Affiliations:** LOMWRU, University of Oxford, Vientiane, Lao People's Democratic Republic

1) We have added a paragraph in discussion to mention the limitations of our study: “Our study has limitations, including patients collected over only one year, not including recent data, and only collecting upper respiratory tract samples. No serology was performed and no published evaluation of the accuracy of FTD33 kit for the diagnosis of *M. pneumoniae* infection is available.”  

 2) FTD33 is a commercial kit, in the validation document provided by the manufacturer only information on analytical sensitivity and specificity is provided - we have included this in the methods section: “validation by the manufacturer showed a detection limit of 100 copies/mL of plasmid and no reaction with *M. pneumoniae*-negative samples by real-time PCR reference methods (communication from Fast-track Diagnostics).”

 3) We did not find any published articles on the evaluation of sensitivity and specificity of FTD33 for *Mycoplasma pneumoniae* detection. 

 4) We added in the discussion that with no published data on the accuracy of FTD33 for the diagnosis of *M. pneumoniae* infection we could have underestimated the number of cases.

 5) All symptoms for the three patients (rigors, sputum, runny nose, sore throat, vomiting, nausea) are consistent with *M. pneumoniae* infection, as it is mentioned in discussion section.

 6) I added one sentence in footnote of the Table 1. *M. pneumoniae* infection can have protean clinical manifestations and therefore we would prefer not to label clinical manifestations as inconsistent.  

 7) If the reviewer is pointing out that these data are part of the larger data set of ARI in Laos, we confirm this but argue that a more detailed report on *M. pneumionae* infection is important on public health grounds to highlight this disease that is neglected in Laos and elsewhere in SE Asia.

- Author response to "Intro" section in Review Report:

1) Thank you for the comment. We replaced *Mycoplasma pneumoniae* by *Mycoplasma* spp.   We have moved the statement “ *M. pneumoniae* has been described from adjoining Cambodia, Vietnam and Thailand, but not, as far as we are aware, from Myanmar/Burma » to the introduction, and we have mentioned in the introduction the fact that only one hospital has the facilities for the diagnosis of *M. pneumoniae* infection and that there is a need for more information on the burden of *M. pneumoniae* infection in Laos.  

- Author response to "Methods" section in Review Report:

1) The writing up of the complete study over several years is in progress. However, all single microorganisms will be not discussed individually, therefore we felt important to publish a separate paper to highlight the importance of *M. pneumoniae* and to raise clinical and public health awareness.  

- Author response to "Results" section in Review Report:

1) We removed the mention of patient gender in that sentence.  Although we cannot 100% rule out contamination, the test were performed following good laboratory practice using quality controls. Those results are likely to correspond to co-detection of several micro-organism (co-infections and/or carriage) in upper respiratory tract as it is reported in other studies. We added this information in the footnote of the Table 1: “Contamination cannot be 100% ruled out, however the test were performed following good laboratory practice using quality controls. Those results are likely to correspond to co-detection of several micro-organism (co-infections and/or carriage) in upper respiratory tract as it is reported in other studies: more than one microorganism was detected in 73% of patients with ARI in the study in Lao by Phommasone *et al*. 202222 and in 93% of cases (hospitalized children with severe pneumonia) in PERCH study ^23^.”  

- Author response to "Discussion" section in Review Report:

1) The detection test used was PCR which is highly specific and sensitive. In addition as *Mycoplasma pneumoniae* isolates from patient samples were not handled in the laboratory, it is very unlikely that those results could be false positives due to contamination. We added this sentence in the discussion.  Thank you for the suggestion. Discussion of these important issues would greatly increase the size of the paper and make it more of a review than a case description from Laos. We would be grateful for the editors advice as to whether expanding the paper is felt appropriate or not. We would prefer to keep it as a case report from Laos.   We think we already have addressed this in the discussion by the following sentence: « Hence, enhanced awareness and routine diagnostic systems for *M. pneumoniae* detection in Laos, and research into the local prevalence of antibiotic resistance in this pathogen, are needed, both for individual patient care and surveillance to understand the epidemiology of *M. pneumoniae* infection and to inform treatment guidelines. »
